# Dominating Risk Impositions

**DOI:** 10.1007/s10892-022-09407-4

**Published:** 2022-10-11

**Authors:** Kritika Maheshwari, Sven Nyholm

**Affiliations:** 1 University of Groningen, Groningen, Netherlands; 2 Utrecht University, Utrecht, Netherlands

## Introduction

Consider two cases, both based on reports from the *New York Times*:

*CARS*: In December of 2018, some residents of Chandler, Arizona, were slashing the tires of, throwing rocks at, and even waving guns at self-driving cars operated there by Google. These residents were protesting experimental trials of self-driving taxi services in their neighborhood in fear that these cars were making public streets unsafe, among other things. For example, Mr. O’Polka – whose 10-year-old son was nearly hit by one of the self-driving cars – complained in an interview, “They say [the cars] need real-world experience, but I do not want to be their real-world mistake.” To this, his wife added, “They didn’t ask us if we wanted to be part of their beta test”^1^

*CORONA*: In March of 2020, janitors working in Amazon’s offices in San Francisco filed complaints against their contractors who, without their knowledge, made them clean offices used by people infected with SARS-CoV-2. While the company went to great lengths to ensure that their tech and finance employees were fully informed and protected against the risk of infection, the janitors were left in the dark. Feeling upset that the company was compromising their safety, the janitors complained that their lives were being valued less than those with resources and power. For example, Ms. Carrion, who faced a similar situation at Citigroup, said in an interview, “I would not have gone for my cleaning shift had I known about my possible exposure to risk”. Worrying that she may have contracted the virus and passed it on to her ill husband, she added, “So who guarantees my safety?”^2^

The risky experiment in Chandler was ultimately put to a halt. Luckily, none of the protestors, including Mr. O’Polka, were materially harmed by the experimental self-driving cars. Likewise, the janitors at Citigroup, including Ms. Carrion, did not get infected by the virus. In both cases, risk-bearers incurred what Judith Thomson (1986) calls “pure risks”, namely, risks that failed to materialize into harm. Not everyone is that lucky, however. In Tempe, Arizona, a woman was hit and killed by an experimental self-driving car, the first recorded case of pedestrian fatality of its kind. In various other Amazon facilities, a number of janitors succumbed to the virus infection and fell seriously ill.^3^

Despite their distinct fates, most would agree that risk-bearers in both lucky and unlucky cases have been wronged by being subject to serious risk of harm, independently of whether these risks materialized for them. What, then, is the basis of their complaint? In other words, what, if anything, makes imposition of risk in cases like *CARS* and *CORONA* wrong?^4^

A natural response is to focus on complaints of our two “lucky” risk-bearers. Paraphrased, risk-bearers are objecting to being exposed to risks they did not know about or agree to, as Mr. O’Polka notes. They are also objecting to the differential care and concern shown by those with resources and power towards their safety, as Ms. Carrion notes. Both concerns are morally salient: they say something about how risk-imposers relate to risk-bearers in imposing risks on them that affect their safety, much against their will, knowledge, and sometimes both.

If we accept that these concerns should inform – at least in some way and to some extent – our understanding and evaluation of why and how risk-imposers act wrongly, then existing accounts in the literature fail to fully capture and acknowledge them. To this end, we propose an alternative. On our view, risk-bearers like Mr. O’Polka and Ms. Carrion suffer a distinct form of injustice that is best captured by what republican theorists describe as the interpersonal or relational harm of domination.

In the received terminology of republican theorists, the condition of being wrongfully subjugated by those who possess some degree of power over us *and* are in a position to exercise it at their own discretion is often called ‘domination’ (Pettit 1997, 2012; Skinner 1998; Lovett 2014). Within this framework, an individual’s freedom from others’ domination “is restricted not merely by actual interference or the threat of it, but also by our awareness of the mere fact that we are living in dependence on the goodwill of others” (Skinner 2002: 247). Thus conceived, to claim that someone is dominated is, among other things, to make a claim about their *status* or social standing as individuals. Domination is a moral harm that, in essence, turns on how people relate to one another; it represents a morally (and politically) problematic relationship between those with dominating power and those subject to it^5^.

In this spirit, we argue that the complaints of risk-bearers like Mr. O’Polka, Ms. Carrion, and others in a relevantly similar situation are best understood as complaints against being subjected to what we call *dominating risk impositions*. Dominating risk impositions are characterized as constituting a problematic form of relationship between those who have uncontrolled or unchecked power to impose risk and those who are vulnerable to its imposition. For their part, risk-imposers act as a kind of domineering master or doorkeeper who can decide, without appropriate constraints or checks, what risk-bearers are safely able to do, whilst the former are often powerless to avoid, resist, or contest the imposition of risk on them.

When the safety of risk-bearers is dependent on or is at the mercy of the risk imposer’s will in this way, it can be enough to make the risk imposition dominating or to make it generate domination-like effects. The relation between Mr. O’Polka and those in charge of the risky experiments and that between Ms. Carrion and her employers are instances of such cases – or so we argue.

We suggest that focusing on freedom as nondomination in capturing cases like *CARS* and *CORONA* is of both theoretical and practical importance. Firstly, the relationship between risk imposition and domination remains underexplored in the literature. If our account is convincing, we would succeed in offering a new explanation for why imposing risk is sometimes wrong. This result should not only interest philosophers of risk, but also republican theorists, as we identify certain instances of risk imposition as a distinct, potential source of domination.

Secondly, our proposal is well-suited to make better sense of a wide range of everyday cases that are structurally similar to our real-life, motivating cases above. For instance, powerful big-data tech companies like Meta (formerly known as Facebook) are increasingly subjecting consumers to risk of privacy theft, whilst effective mechanisms that require such powerful agents to be held accountable by risk-bearers are still lacking (Veliz 2020). Similarly, oil and gas industries operating in regions of Oklahoma and Groningen are battling lawsuits for imposing grave risks of livelihood damages and losses to local residents as a result of years of extensive fracking without their agreement.

For years, these companies have been found guilty of freely profiting from gas drilling without regard for the risk of earthquakes and damage they burden residents with, whilst ignoring the interests of residents in their decisions to drill, regulate or compensate for harms (Mallor 2019). Similarly, after years of world-wide protests, the biotech company Monsanto was ultimately found guilty of knowingly subjecting farmers to the risk of poisoning from toxic pesticides and using their power to hide information about the risks posed by their products (Roeser 2018). Such examples of powerful risk-imposers subjecting vulnerable risk-bearers to serious risks with impunity are widespread and fall within the explanatory scope of our account.

Our discussion develops in the following parts. Section 2 surveys recent accounts from the growing literature on the moral significance of risk imposition, focussing on their treatment of two ‘stock examples’. Drawing on their insights and shortcomings, we offer the Domination Account as an attractive development of the existing accounts in Sect. 2. In Sect. 4 we discuss republican reasons for taking instances of dominating risk imposition seriously. In Sect. 5, we address some potential objections. Section 6 concludes.

Before proceeding, two quick clarifications are in order. First, a terminological point. The word “risk” is used in a variety of ways, from everyday language practices that indicate potential danger to more technical notions that require extensive calculation (Hansson 2003). For our purposes, we employ a fairly broad notion of risk and risk imposition. To impose risk, as we understand it, is simply to create or raise the likelihood of something bad happening to someone else^6^. Second, in making our case, we focus on inter-personal cases involving individual, adult agents in the role of risk-imposers and risk-bearers, for simplicity. The discussion, however, is meant to be relevant and applicable to cases involving groups or institutions and cases where risks are imposed systemically or structurally by coordinated or disparate actions of many individuals.

## Existing Accounts: A Dialectical Exposition

We now turn to discussing recent proposals from the literature. These are: the Interest (James 2016), Contractualist (Kumar 2009), Autonomy (Oberdiek 2012, 2019), and Negative Freedom (Ferretti 2016) accounts. There is a broader range of accounts on offer than the ones we discuss here (Hayenhjelm and Wolff 2011)^7^. But we limit our focus to these for three main reasons.

First, these accounts aim to capture the distinctive moral significance of pure risk impositions *as such*, or more generally. Their appeal as explanatory accounts thus hinges – at least to some extent – on whether they can also satisfactorily capture specific real-life cases like *CARS* and *CORONA*.

Second and importantly, all four accounts belong to a family of views that offer a non-consequentialist explanation of what is wrong with imposing risks on others. That is, they argue – albeit in different ways – that the moral problem with imposing risk is at least partly relational; sometimes it involves treating risk-bearers wrongly, over and beyond how risk affects the welfare (or well-being) of the risk-bearers.

And third, understanding the insights of these accounts will help to set the stage for our own view. In other words, discussing these accounts thus helps to situate our own account insofar as we offer an alternative description of the relational wrong of imposing risk on others in terms of treating others in a dominating way^8^. We hope to show that intuitions driving these accounts can be systematically captured and defended within a single theoretical framework of republican freedom as nondomination.

Notably, discussions of these accounts primarily focus not on specific real-life cases, but rather on what we can call two ‘stock examples’ of risk imposition. These are^9^:

### RECKLESS DRIVER

You are out driving in a careful way, making sure that you and others on the road interact with each other in a safe and well-coordinated way. Suddenly, a reckless drunk driver comes driving at a high speed, making life less safe for everyone.

### RUSSIAN ROULETTE

Your friend decides to play Russian roulette on you while you are sleeping so he can enjoy a few moments of sinister fun. Luckily for you, when he pulls the trigger, nothing happens. You never learn about what he did.

While distinct non-consequentialist accounts differ in their approaches, they frequently aim to articulate what is morally problematic about risk-imposers conduct by appealing to these two examples.

### The Interest Account

In discussing *RUSSIAN ROULETTE*, Aaron James (2016: 5–8) notes that you have a complaint against your friend pointing a loaded gun at you insofar as in doing so, he substantially raises your *ex ante* prospects of being harmed. To capture the basis or grounds of your complaint, James focuses on the issue of control. He claims that individuals have a basic, objective interest in avoiding exposure to risk in certain areas of their lives, “especially when their exposure is not in their personal control” (James 2016: 6)^10^. As James further notes,

“One way of respecting this interest is to give those affected a suitable opportunity to control their level of exposure to the relevant risks (you are left free to put the gun to your own head, or not, without my encouragement)” (James 2016: 6)

James’ interest-based account, then, highlights the significance of the risk-bearer’s control, or lack thereof, over the risks that others expose them to. At the same time, his discussion also raises rather than answers an important question: What about the control exercised by the risk-imposer, instead of the risk-bearer, when they play Russian Roulette on them? Whilst you may enjoy the freedom to play Russian Roulette with yourself such that whether you live or risk dying is under your control, it is problematic that *someone else* exercises similar control over you. This clashes with your interest in being in control over what risks you are exposed to.

### The Contractualist Account

In discussing *RECKLESS DRIVER*, Rahul Kumar (2009) makes a similar suggestion. As Kumar writes,

“…one has been wronged by the drunk driver…simply in virtue of his

having, without justification, taken your life in his hands by exposing you, even briefly, to so serious a risk. An adequate analysis of being wronged ought to be able to make good sense of our intuitions in this kind of case…” (Kumar 2009: 103).

Kumar’s description of drunk drivers’ risky conduct being akin to “taking another’s life in one’s hand” is another way of noting that there is something problematic with risk-imposers exercising control over others in subjecting them to risk. But Kumar also seeks to embed this observation into a contractualist framework. To this end, he offers the following contractualist characterisation of how and why the drunk driver *wrongs* the pedestrian: the drunk driver fails to comply, either intentionally or negligently, with a legitimate expectation that the pedestrian is entitled to have, namely, a reasonable expectation that any driver “drives in a manner conducive to keeping the risk within certain acceptable limits” (Kumar 2009:107)^11^. The wronging thus consists in the particular way in which the risk-imposer relates to the risk-bearer and what they can legitimately expect of others. In culpably failing to keep the risk within certain acceptable limits that the risk-bearer could reasonably agree with, the drunk driver fails to appropriately recognize her status as a person who is entitled to, say, high levels of safety on public roads.

Yet, notably, the fact that the risk-imposer’s conduct involves “taking another’s life in one’s hand” is still not part of the contractualist explanation of why exactly, in doing so, he wrongs the pedestrian. If our explanatory goal is, to put it in Kumar’s words, to make good sense of our intuition in this case, then we need to provide an adequate analysis of what, in addition to wrongly imposing risks beyond acceptable limits of what is allowed under a contractual relation, makes exercise of control over another’s exposure to risk wrong.

### The Autonomy Account

John Oberdiek (2009; 2012: 342–356), though himself not a contractualist, broadly agrees with Kumar. Here is how he summarizes his development of what he takes to be the essential moral insight in Kumar’s reasoning:

“…The idea, at bottom, is that imposing risk on others is morally significant

…because risk impositions amount to [1] claims of authority over others lives and, more specifically, [2] over the range of options that constitutes

their autonomy…” (Oberdiek 2012: 355).

Oberdiek makes two claims here, as indicated by our numbering. The first claim attends to Kumar’s suggestion of taking another’s life into one’s hand by noting that wrongful risk imposition “involves acting on the mistaken presumption that someone else’s authority [over their life] is one’s own to wield” (Oberdiek 2009: 391). The second claim adds that the risk-imposer’s mistakenly self-attributed authority is exercised over the risk-bearer’s autonomy. The notion of autonomy appealed to by Oberdiek is the one developed by Joseph Raz (1986), according to which,

“For a person to enjoy an autonomous life he must actually use [his rational] faculties to choose what life to have. There must in other words be adequate options for him to choose from. Finally, his choice must be free from coercion and manipulation by others, he must be independent.” (Raz 1986: 373).

To understand the effect of risk on one’s autonomy, Oberdiek offers the following analogy: think of it as someone laying death traps in your way (Oberdiek 2012: 375). Just as there are fewer adequate options before you when you are confronted with a death trap, imposing risks on others has similar effect of reducing the full range of choice-worthy, safe options required for meeting the conditions of autonomy^12^.

Oberdiek’s autonomy account makes headway towards capturing the risk-imposer’s exercise of power or control as being explanatory central to evaluating his wrongful conduct. Yet, it is still importantly open-ended when it comes to explicating precisely what is it about the risk-imposers’ wielding of power that makes risk-bearers victims of moral wrongs or injustice.

Moreover, in cases like *CARS* and *CORONA*, risk-imposers exercise power that is, problematically enough, theirs to wield. For instance, Ms. Carrion’s objection against her employers is rooted in the fact that they actually occupy positions of power or authority allowing or enabling them to subject her to risks, rather than merely acting on the *presumption* that they have such power, *contra* Oberdiek. Thus, for our purposes, Oberdiek’s discussion opens up an important question: what is wrong about risk-imposers wielding this kind of power over risk-bearers when such power is problematic, and why exactly is it morally problematic?

### The Negative Freedom Account

When Maria Paola Ferretti (2016: 266) discusses the autonomy account, she suggests that Oberdiek’s focus on risk’s impact on one’s options is a step in the right direction. However, she proposes shifting the focus away from Raz’s concept of autonomy and instead draws a link between risk and the risk-bearer’s freedom, where freedom is understood as an option or opportunity concept. On this account of freedom, an agent’s freedom is understood in terms of the absence of “external impediments” to options, so that the extent of an agent’s freedom is determined by the number of different options that are available to her.

Accordingly, Ferretti proposes conceptualizing imposition of risk as a form of *actual* interference with the full range of options open to an agent. She writes,

“…what counts, really, are external impediments to options. . [W]hat is wrong with risk imposition is that it negatively affects the opportunities open to a subject. This notion is captured by economists and social choice theorists using the concept of an opportunity set, that is, a set of available alternatives from which an individual can choose an element…” (Ferretti 2016: 267).

For Ferretti, risk-imposers wrong risk-bearers when in imposing risks on them, they reduce their overall negative freedom by constraining the number of set of *compossible* options open to them^13^. While it may appear as if Ferretti’s focus is solely on outcomes, she does not intend her account to be understood as a welfare-focused consequentialist account of what is wrong with risk imposition. Instead, Ferretti presents her account as a broadly Kantian view, and explicitly claims that in order to understand what is morally problematic about risk imposition, we should ask whether imposing risks is compatible with treating others as moral agents (Ferretti 2016: 262). On this account, respecting others as moral agents requires respecting a certain measure of overall freedom that we owe them.

At this juncture, we can observe an interesting development in the dialectic of these accounts so far. The accounts put forth by James and Kumar rightly highlight the intuition that risk imposers ‘take control over’ and ‘exercise power over’ the risk-bearers, but only go so far in capturing its significance. While Oberdiek’s autonomy account retains this idea to some extent, Ferretti’s focus on overall negative freedom seemingly loses sight of the intuitions regarding exercise of power driving the other accounts.

On the one hand, we grant, following Ferretti, that risk imposition sometimes constitutes actual interference with freedom and, to that effect, constitutes disregarding others as moral agents who should have a wide range of options open to them. On the other hand, we think that in focusing merely on the *actual* impact on one’s options, the moral significance of the risk-imposer’s exercising their (presumed) authority by way of unjustifiably taking control over risk-bearer’s options is lost along the way. The question, then, is whether what gets lost along the way is crucial to explaining what is morally problematic about risk-imposers’ conduct in cases like *CORONA* and *CARS*, and if it is, then in what ways? As we see things, the question, in part, is whether the conception of freedom appealed to by Ferretti is the best one in this context.

## The Domination Account

In her discussion, Ferretti appeals to the work of liberal thinkers who conceptualize freedom in terms of non-interference with one’s options (e.g. Carter 2003; Steiner 1994; Cf. Sudgen 1998). Some liberal theorists argue that being free is a matter of having a range of options in the absence of any actual interference (Berlin 1969)^14^. By contrast, republican theorists argue, *contra* liberal theorists, that having a range of unconstrained options is insufficient for freedom. Instead, they propose an additional condition, namely, that one should have a range of options independently of other people’s will over whether one has those options.

In spelling out this idea, republicans appeal to a number of paradigmatic cases. For instance, consider an enslaved person whose ‘kind master’ allows him to do whatever he wants (cf. Pettit 2012). What republican theorist find distinctively problematic is that the master could easily interfere and change what the slave is able to do. Republicans emphasize that even if the probability that the kind master interferes with the enslaved person is low, it is objectionable for the master to have the *capacity* to setback or interfere with his range of options. This is an example of a structurally based social relationship that puts the enslaved at constant risk of interference.

Another example is that of a gunman or a bully who stops a passer-by, draws a pistol, and makes the demand ‘your money or your life’ (Skinner 2008: 95; Blunt 2015). In doing so, he interferes with the passer-by such that the passer-by must obey the gunman’s command if he wishes to save his life. Being subject to the threat of ‘your money or your life’ renders the passer-by’s fate at the mercy of the gunman (Gädeke 2020). This is an example of a form of interactional, episodic exercise of arbitrary power. The gunman’s power establishes a temporary social relationship, which the passer-by cannot exit or contest without risking high costs like losing his life or his money (Laborde 2010: 57).

The republican connection between social relationships and freedom distinguishes it from liberal theories of freedom of the sort Ferretti appeals to in her theory in the following way. While the latter exclusively focus on an individual’s range of unconstrained options, the former type of theory focuses on, among other things, the status, protections or assurances, and interpersonal relations of the individual with others. In particular, for republicans, what matters most is the ‘condition suffered by persons or groups whenever they are dependent on a social relationship in which some other person or group wields arbitrary power over them’, regardless of whether they exercise this power (Lovett 2010: 20). They identify this condition as the condition of being *dominated* or being unfree.

Republican freedom, then, is freedom from domination. Admittedly, domination is itself a contested concept with various competing accounts on offer. In what follows, we will mainly draw on insights from Philip Pettit’s (1996, 1997, 2012) relational conception of domination^15^. While we don’t here defend Pettit’s account of domination against existing alternatives, we follow Pettit’s lead primarily for the reason that his account is one of the most well-developed and widely influential accounts in contemporary discussions of domination^16^.

According to Pettit (2012), an agent’s choice with respect to her options is free, or non-dominated, if she has the capacity to choose between the options and no other agent has the unchecked, uncontrolled, or arbitrary capacity to interfere with her choice. By contrast, she is unfree or dominated in a certain choice by another agent or agency if and because the latter has a capacity or the power to interfere with her choices that is arbitrary.

What makes an interference arbitrary, and hence an instance of dominating power? Arbitrariness has been interpreted in different ways by republicans (Lovett 2012). Pettit (2012: 57–58) explicates arbitrariness in terms of control or, rather, the absence of it. Power is arbitrary when those subjected to it do not have control over its exercise (Pettit 2012: 50). To be free or non-dominated in the republican sense, then, requires the absence of uncontrolled, or unchecked, or arbitrary power. That is, power that is exercised at will and with impunity.

The with-impunity condition holds that there is no (significant) penalty, loss or difficulty faced by the dominating party for their interference. They enjoy *carte blanche* to the extent that they do not have to justify themselves to their victims or to others. The at-will condition, by contrast, holds that they can initiate interference at their own pleasure – at their own whim, without some independent legitimating circumstance. When these two conditions are realized – to some extent and degree - then the capacity for interference that they enjoy amounts to arbitrary or dominating power (Pettit 1996). There is support for the claim that both of these conditions are fulfilled in the case of risk-bearers like Ms. Carrion and Mr. O’Polka^17^.

Take *CARS* for instance. The incident with Mr. O’Polka and fellow residents took place in the American state of Arizona where self-driving car companies like Uber and Google started running experiments to test fully driverless cars. These companies shifted their testing grounds from states like California to avoid stricter safety regulations to Arizona that is famous for its lax regulation and industry-friendly policies that allow companies to avoid various legislative hoops (for instance, not requiring them to obtain certain special safety licenses) and prioritize developing their products in a competitive market as fast as possible.

In all this, those who are potentially most immediately affected by these decisions, namely, at-risk residents like Mr. O’Polka are excluded from participating in, or being represented in, the decision-making process not only by those who are designing and testing these technologies, but also those who are approving its testing. For instance, there are no procedural or legal requirements for including or consulting risk-bearers in the decision to operate risky driverless cars in their neighbourhood. Such decisions are made at the level of state policy and are usually informed by the states’ and companies’ economic and technological interests, rather than the interests of individuals who do not wish to and do not approve of being made subject to risks by getting into accidents with driverless cars^18^.

On the one hand, the highly technical, political, and legal nature of such decisions act as barriers for individuals to exercise any oversight in assessing and opposing the risks they are subject to. On the other hand, risk-imposers enjoy full control and discretion over such decisions given their (apparent) role and position as experts and decision-makers. From the risk-bearer’s perspective, then, the risk-imposer is a person in the role of a domineering master or a bully, so to speak. In imposing risks on others, they act as if they have a right to make decisions about which safe options should be open to the risk-bearers. Doing so is morally objectionable insofar what happens to risk-bearers is dependent on, or at the mercy of the risk-imposers’ will.

To draw a contrast, consider the difference between exposing oneself to the risk of getting infected with some virus because one is careless or negligent, versus being exposed to the same risk because your boss enjoys unilateral decisional authority over whether or not to implement precautionary safety measures that protect you from the risk, as in the case of Ms. Carrion. Or compare the difference between exposing oneself to the risk of getting into a car accident because one is driving recklessly and being exposed to the same risk because a big corporate company controls whether or not to conduct risky experiments involving potentially unsafe self-driving cars while you lack any say, as in the case of Mr. O’ Polka.

In these contrasting cases, when your exposure to risk is voluntary, the interference you face with respect to the options is non-dominating. This is so because when you take the risks on your own terms, you control – to some extent and degree – how the risks interfere with your options. By contrast, when risk-imposers subject somebody like Mr. O’Polka to these risks, they interfere with them to the extent that *they* control and decide what risks others (viz. Mr. O’Polka) are exposed to – with impunity and at their own whim. This is the core insight of what we call the *Domination Account* of morally problematic risk impositions.

On the *Domination Account*, imposition of risk is dominating, or has domination-like effects, when and because risk-imposers’ imposing risks on risk-bearers makes the risk-bearer and their safety dependent on the risk-imposer or is constitutive of risk-imposers’ exercising their arbitrary power over the range of safe options that risk-bearers are in a position to choose from. There are two important points to note about this account. First, the account does not hold that risk imposition is always dominating. Rather, the idea is that one key aspect of what makes an important class of wrongful instances of risk imposition morally problematic is that they involve a dominating form of relation or interaction between the risk-imposer and the risk-bearer^19^. Second, our account does not require that one be subjected to risks multiple times or frequently for risk imposition to create a dominating or domination-like relation. A terrorist, for instance, needn’t repeatedly or frequently expose a group to the risk of losing their lives to qualify as imposing risk in a dominating or domination-like way.

Sometimes, however, it might be that risk-bearers may become merely *vulnerable* to domination in the future because some instance of episodic risk imposition is itself insufficient to create something that is severe enough to count as a form of domination. In other cases, it might be that the risk-bearers’ vulnerability to domination *viz-a-viz* risk imposition arises, in part, due to them already standing in a pre-existing relationship of domination with others. In such cases too, our account nevertheless holds that the moral problem with subjecting others to risk is one of domination insofar risk-imposers have the capacity to exercise their arbitrary power over risk-bearers and affect what they can safely do^20^.

On this account, then, the relation of domination implied by risk imposition is simply that there is domination or domination-like effects *when* imposing risk on others makes one party dependent on the other, when it reinforces such dependencies, or when it involves or constitutes exercise of arbitrary power on behalf of the risk-imposer. Focussing on domination thus properly orients our concern towards the power dynamics between risk-imposers and risk-bearers by explicating it in terms of the latter’s dominating control or power over the former’s options. This seems to be a large part of what some existing accounts, such as those offered by James, Kumar, and Oberdiek are trying to capture and what seems to potentially get lost in Ferretti’s translation of their views into a view expressed in terms of the liberal notion of option freedom as non-interference. Considered in this way, the Domination Account is not meant to replace these aforementioned existing accounts. Rather, it is meant to complement or build on their insights.

We suggest, then, that the republican conception of freedom as nondomination is the best way to capture and articulate the moral complaint against risk-imposers’ conduct based on their way of relating to risk-bearers when subjecting them to what we call “dominating risk impositions”. Importantly, dominating risk impositions share three core features that are characteristic of cases used for theorizing about domination. These features are power differences, dependency, and arbitrariness^21^. We briefly consider them in turn below.

### Power Differences

A constitutive feature of dominating risk imposition is the relation of power between risk-imposers and risk-bearers. In *CORONA* and *CARS*, when risk-imposers expose others to a serious risk of harm, they exercise power over risk-bearers wherein the latter often lack the same degree or extent of power over the former. For instance, Ms. Carrion lacks the power to subject her contractors to the risk of getting infected, at least without incurring heavy costs such as losing her job. The same is not true of her risk-imposers. When risk-bearers lack reciprocal power over risk-imposers, there is an imbalance of power.

Does this mean that risk imposition is dominating only when agents stand in relations of unequal power? Not necessarily. For instance, in the drunk driver case, the exercise of power may happen between two equals: you can, if you wish, expose the reckless driver to the same risk of harm. Or we can imagine a situation between two reckless drunk drivers, each exposing one another to risk of harm, and taking each other’s lives into their respective hands. The fact that one drunk driver exposes the other to the same risk, then, may not necessarily be explained by status inequalities. There might be none.

Yet, the mere fact that the reckless driver is able to, and in fact does, take your life into his hands and subjects you to risk at his own will, and vice versa, suffices to mean that he dominates you. We can call this dominating risk imposition between equals^22^. The risk imposition is symmetric or reciprocal. By contrast, in our real-life cases, the risk imposition and exercise of power happens between unequals. The risk imposition is asymmetric or non-reciprocal^23^. We can call this dominating risk imposition between unequals.

Note that the *Domination Account* also allows for different ways of exercising one’s dominating power. A risk-imposer may do so negligently, recklessly, intentionally, or by way of deceiving, coercing, or enticing the risk-bearer in ways which are decisive in changing what the latter is able or wants to do. For instance, in *CARS*, when Mr. O’Polka is exposed to a risk of accidents, Waymo’s interferences with his options stem from their negligent failure to observe their duty of care towards him. In doing so, they replace his option to walk safely in his neighbourhood with a riskier option.

Similarly, in *CORONA*, when Ms. Carrion’s employers fail to inform her of her potential exposure to risk, they interfere with her options negligently, blinding her to the true nature of her available options to work safely by affecting their expected outcome. In both cases, whilst the particular ‘form’ of power involved in exposing others to risk may differ, the basis of one’s objection to the imposition of risk remains the same, namely, that this power is exercised in a *dominating* manner or mode^24^.

### Dependency

Domination involves a form of dependence (Skinner 2008). To have power over someone often entails that they cannot easily exit the relationship or interaction without prohibitive exist costs (Lovett 2012). For instance, the enslaved person cannot exit their relationship with the master, because the costs may involve them getting mistreated by their master. By contrast, for someone who depends on their boss for their income but could easily get another job, the exit costs are comparatively low. Their dependence may perhaps be too low for domination to occur.

There are many instances of everyday risk impositions that involve varying degree of dependency. For instance, if you want to be a good swimmer, you are dependent on your coach as they can expose you to some risk of harm when they teach you how to swim. However, such dependency entailed by the kind of risk and how much risk they can expose you to (not too much to actually cause you harm but enough to make sure you learn) does not necessarily make their risk imposition dominating if, for instance, you can voluntarily choose a different coach without incurring significant costs.

Similarly, whilst driving on public roads, people depend on each other insofar some risk impositions are inevitable (no matter how many precautions you take, there is always a chance that someone will crash into your car). As is widely noted, driving, as a social activity, is made possible in virtue of drivers (non-drivers, and pedestrians) being dependent on each other to impose small risks and taking appropriate precautions (Hansson 2003).

By contrast, when we look at a case like the reckless driver, your dependency is heavily dependent on what he does. Similarly, in case of Ms. Carrion, whether or not she gets infected with the virus, and consequently poses risks to others, highly depends on how her contractors act. The dependency is significant as it may cost her her life, job, and health. This dependency is made even more problematic by the fact that her employers are (legally) responsible for providing her with a safe working environment. In such cases, risk-bearers face a double-edged sword: those who are responsible for their protection and safety are also the ones who have the power to take it away, or to threaten it by imposing risks on them.

### Arbitrariness

Finally, the third constitutive feature of dominating risk imposition is that the power risk-imposers hold over risk-bearers is arbitrary. As noted, on Pettit’s account, when power is arbitrary, it is unconstrained or uncontrolled by those subject to it. Arbitrariness is a matter of degree (Pettit 1997: 74–77); how free one is is determined by how many non-dominated options one has and how important they are (Schmidt 2018). Moreover, how intensely or how strongly risk-bearer’ options are dominated or non-dominated is in turn a function of a number of things.

For instance, it may be determined by how many and which options are subject to risk-imposer’s arbitrary interference as well as how far their power is controlled or uncontrolled depending on the reliability, effectiveness, and accessibility of resources and safeguards that protect risk-bearers from risk-imposers’ dominating power. Accordingly, how unfree risk-bearers are can vary along (at least) two dimensions, namely the extent and intensity of dominating risk impositions. This indicates that the issue of the dominating effects of risk impositions is not an all-or-nothing matter; some impositions can be more dominating whilst others less so.

## Taking the Domination Account Seriously

The Domination Account makes headway in existing discussions of the ethics of risk imposition in a number of ways. We will here consider three points.

Firstly, this account is particularly well-suited to capture cases of wrongful *pure* risk impositions where the risked outcome doesn’t materialize. On this view, what matters is that someone stands in a relation with you where they have the *capacity* to interfere with your options, irrespective of whether the risk materializes. The problem of domination still persists even if the risk-bearers ultimately remain unscathed from the possible outcome of the risk, or any psychological effects that being exposed to risk has on them.

Secondly, recall that the accounts discussed earlier also identify the wrong of imposing risks on others in relational terms, where this pertains to disrespecting them as moral agents. On the one hand, the Domination Account is compatible with this understanding of relational wrongness insofar domination restricts the extent to which individuals can live in a relationship of mutual respect with one another. On the other hand, it also adds to our understanding of this relational wrong by capturing it in terms of risk-imposers’ creating asymmetric dependencies and holding power over and relating to risk-bearers in a way that affects the latter’s *status* or social standing as free individuals living in a community with others.

Thirdly, many accept that the social relations within which risks are distributed, exchanged, shared, or taken play a role in the acceptability of certain societal risks (Anderson 1988). Yet, most existing accounts of the wrongness or impermissibility of imposing certain risks on others don’t explicitly consider the significance of social relations or the differences in power dynamics that hold between risk-imposers and risk-bearers. As our discussion makes clear, any imposition of risk that is constitutive of establishing or instantiating a dominating relationship is likely to render such risk imposition morally impermissible, independently of any other moral considerations, such as any benefits, involved. Besides these reasons, there are two features of dominating risk imposition in particular that are worth taking seriously, which we will now discuss.

### Modally Robust Interference

For most republicans, whether an individual is free does not depend on whether or how much someone *actually* interferes with their options. Rather, the mere uncontrolled power to interfere as such can render someone unfree, even if the probability of such interference is low or negligible because the dominator is not strongly disposed to exercise their power. By contrast, one is unfree in the liberal sense if and when someone actually interferes with one’s options. Some authors explain this difference between the republican and liberal views in terms of modal robustness (List and Valentini 2016; Pettit 2012; Schmidt 2018).

For instance, to be free in the republican sense requires that one has a set of freedoms F independently of another person’s will across a set of nearby possible worlds in which the other person changes her will over whether or not you should have F. In case of the kind master, the enslaved person’s options for action are not modally robust or are only weakly robust because she lacks F in nearby possible worlds, in which the kind master changes her mind and decides that she should not have any option-freedom. Not so for liberal freedom. On that view, one is unfree or lacks F when one lacks these freedoms in the actual world.

It is this difference in modality that brings out a distinctive republican account of the moral significance of imposing risks and sets it apart from Ferretti’s negative freedom account as well as from Oberdiek’s autonomy account considered earlier. Recall that in *CARS* and *CORONA*, risk-bearers not only objected to a specific instance of being subject to risk; they also feared the *possibility* of being exposed to risks in the future. For instance, in asking, “So who guarantees my safety?”, Ms. Carrion voices her lack of assurance that she will not be interfered with again in this way by those who have control over her risk exposure.

Although the risk-imposers may have no immediate interest in or need to impose risks, risk-bearers are constantly at unease insofar they might find that their options have been changed for the worse should the risk-imposers change their mind. The *Domination Account* translates these concerns into concerns about unfreedom. Even when the dominating risk-imposer does not actually interfere with one’s options, the *mere possibility* of being subject to risk by them renders risk-bearers vulnerable. This is because when risk-bearers face dominating risk impositions, their options lack robust protection in close possible worlds as their options can be taken away or made unsafe at any moment.

Accounts that focus on liberal freedom or autonomy do not automatically capture this, insofar they only focus on *actual* exercises of power and ignore the various effects that *potential* exercises of a risk-imposer’s dominating power may have on the risk-bearer. By contrast, the *Domination Account* recognizes that there is more to the ideal of freedom than merely being “left alone”. What matters when it comes to threats to one’s freedom is not just that one *in fact* suffers a reduction in options when one is subject to risk, but that one *could* be due to the power that others may have over one.

Individuals not only want their safe options to be protected from actual interferences; they also want this protection to be modally robust for a number of reasons. For instance, even if the likelihood that risk-imposers subject them to risks remains low, the uncertainty of potentially being interfered with can foster feelings of fear, distress, vulnerability, insecurity, and helplessness, amongst others. As Elizabeth Cripps (2013) notes, “living with risk can also lead to a more general paralysis of the will, so that even in those other areas in which it would remain perfectly rational to form plans, individuals feel unable to do so.” (Cripps 2013: 42).

Apart from affecting their planning agency in this way, being subject to dominating risk impositions can also undermine the risk-bearer’s own sense of self-respect and agency insofar their life depends on the disposition of their risk-imposers and they cannot see themselves as “authors of their own life”. The more risk-bearers depend on someone else’s will, even if they never exercise it, the less control they have over their own life. In contrast, our attitude towards voluntary risk-taking, such as when you play Russian Roulette with yourself, is often tied to our belief or feeling that we exercise control over the risks we have chosen for ourselves.

As Andreas Teuber notes, “we may indeed underestimate our chances of survival in situations where we believe we have some degree of control over the risks we bear, but we also value that control” (Teuber 1990: 248). The value of controlling one’s exposure to risk can be intrinsic, for instance, when it reflects the idea of living life on our own terms. It can also be instrumental, for instance, when it allows us to improve our prospects for attaining certain goods or benefits or developing certain skills and talents.

### Ingratiation

Besides modally robust interference, another distinctive feature of dominating risk impositions is that of needing to engage in ingratiation or other potentially costly or humiliating precautions. In some cases, risk-bearers may be forced to ingratiate themselves to others in response to being subjected to risks at their hands. This aspect of their experience resonates with paradigmatic examples of domination whereby one can never be sure about how and when dominators exercise their power.

For instance, an enslaved person with a kind master might get very good at predicting his master’s behavior, but still lacks assurance that the master won’t switch from being benevolent to being a tyrant. This can have constraining effects on the enslaved person, pushing them into servility to pre-empt or avoid triggering any interferences from their master. They are forced to constantly modulate their behavior to avoid sanctions (Skinner 2008). Similarly, in cases of dominating risk impositions, as long as there is a live or active threat that risk-imposers *could* easily interfere again, risk-bearers are forced to be on a constant “look out” for themselves.

Accordingly, risk-bearers are not only burdened with the anticipation of whether the risked harm will materialize for them; they are also burdened with the anticipation of the further possibility of being exposed to risk (along with anticipating its subsequent consequences for them). This may encourage them to shift or change their behaviour, often unwillingly, to evade or predict being subject to risk in the future. Besides, they may also have to take drastic and costly precautionary steps to ensure that the risks they face don’t materialize for them.

As Jonathan Wolff and Avner de-Shalit note: “…someone who fears being attacked on the street, and so has insecure bodily integrity, may choose always to travel by taxi, and suffer the financial costs, or simply not go out, and lose many opportunities as a result…” (Wolff and de-Shalit 2007: 69). That no actual harm materializes for them and the risk remains a “pure” risk may sometimes depend, in part, on how risk-bearers ingratiate themselves or “get out of the way” in distinct ways to prevent or dodge the risks from being materialized.

To this end individuals may be forced to take morally costly or even desperate steps, such as engaging in violence, as seen in the case of risk-bearers in *CARS* who took to the streets, started slashing tires of self-driving cars, and risked imprisonment to protest the risks they were subject to. At other times, individuals may be forced to simply oblige insofar as strategies or plans for avoiding risks involve heavy exit costs, as in the case of the risk-bearers in *CORONA,* who continued working in risky conditions in fear of losing their jobs or facing retaliation from their managers.

In such instances, exit costs may be high for several reasons; economic or moral costs may be attached to risk-bearers taking precautionary measures for avoiding exposure to the risks, or the attempt to prevent the risks from materialising. Sometimes, it may even require taking on more risks. For instance, you may have to drive very recklessly yourself in order to swerve away from the reckless driver’s car or quit your job if you were in Ms. Carrion’s shoes or risk indictment like in the case of Mr. O’Polka. Besides this, risk-bearers may additionally bear the materialized consequences of the imposed risk itself (e.g., actually getting into a car crash, or actually getting infected and falling ill).

These distinct ways in which risk-bearers are disadvantaged by facing dominating risk impositions gives us reason to take the Domination Account seriously in our ethical thinking in a number of ways. For instance, with our discussion in mind, we can rethink Sven Ove Hansson’s (2003) famous *exemption problem* – that is, the problem of finding criteria for permitting one’s right not to be exposed to risk to be overridden, so that others are allowed to expose one to risk. Hansson himself discusses a number of criteria and advocates that exposure of a person to a risk is acceptable if and only if it is part of a social system of risk-taking that works to the risk-bearer’s advantage. To this, we can add that exposure of a person to a risk is acceptable if and only if it is part of a social system of risk-taking or a personal interaction that does not constitute or involve domination.

More substantively, compensating risk-bearers in cases where they have been wronged by dominating risk imposition is another way forward. Merely offering freedom-enhancing options as compensation as Ferretti (2016: 274) suggests is not necessarily sensitive to the domination or dominating effects they experience. Nor do they undo, rectify or redress the associated harm or damage. Moreover, risk-bearers may continue to be dominated even upon receiving monetary compensation or upon being offered new options that, in principle, increase their overall negative freedom.

The Domination Account thereby forces us to think of different compensation mechanisms and schemes that directly deal with and address the underlying dependencies and power differences between risk-imposers and risk-bearers. Moreover, it also forces us to think of different ways in which we might reduce or eliminate a risk-bearer’s vulnerability towards being subject to dominating risk impositions. On Pettit’s account, we can reduce or eliminate domination by improving an individual’s capacity for action by ensuring what he calls anti-power against others (Pettit 1996). Having anti-power or the capacity or ability to control one’s own destiny implies having sufficient resources and protections in place, which provide individuals with some degree of resilience against possible domination.

To this end, risk-bearers can be offered control or a role in the decision-making process regarding what risks they are exposed to. Republican theorists like Pettit consider participation of affected parties in deliberative procedures as being valuable for its role in rendering the exercise of power non-arbitrary and thus non-dominating. In particular, including risk-bearers in decisions regarding their exposure to risk decreases the chances of risk-bearers being treated merely as subjects of decisions made by risk-imposers. It offers risk-bearers at least an opportunity to challenge or contest the imposition of risk, and allows them to put forward their own proposals, concerns, or demands if and when they disagree as well as to hold risk-imposers accountable for their actions in the event that they act against the wishes of the risk-bearers, amongst other things. (Cf. Roeser 2018: Chap. 6)

Of course, what such a deliberative process would look like in a particular case, and whether it will in fact ensure non-arbitrary imposition of risk is a further question, which is beyond the scope of this paper. However, for now, we would like to simply point out that the Domination Account opens up the space for considering deliberative procedures as one potential way forward to ensuring that risk-imposers avoid subjecting others to dominating risk impositions– to some extent and degree^25^. Besides this, from a republican point of view, it is equally important to focus on enforcing the right kinds of institutional set-ups, procedures, or mechanisms that may check the power held by those in positions of imposing risks, which may go some way to towards remedying the harm suffered and also prevent potential future interferences.

## Objections

Before concluding our discussion, we will now address three potential objections to the Domination Account.

### The Chicken and the Egg Problem

One might question whether our account succeeds in showing that there is something distinctively wrong about imposition of risk *per se* in our motivating real-life cases. One reason is as follows. For instance, consider our analysis of *CORONA*. The case is illustrative of impositions of risk that occur within the context of an employee-employer relationships. Republicans are of the opinion that such ‘workplace’ relationships are often dominating due to structural hierarchy, the nature of working contracts, the general socio-economic position workers occupy as part of an exploitative capitalist system, and so on (Anderson 2017).

One might say, then, that for workers like Ms. Carrion, being wrongfully subject to risk of getting infected at her workplace is an instance of dominating interference. Such interference is dominating *because* it is part and parcel of the *dominion* already exercised by some employers over their employees. Accordingly, if what we’ve called dominating risk impositions occur within already existing relations of domination, it may not be risk imposition *per se* that seems problematic in and of itself, but the relations of domination within which such risk impositions take place.

This objection forces us to reconsider the order of explanation that the Domination Account offers: are certain cases of risk imposition dominating, and thus wrongful, because they are part of interactions between agents already entangled in relations of domination; or are agents dominated or find themselves in relations of domination when and *because* the wrongful imposition of risk is itself dominating? If the former picture is correct, then risk imposition is sometimes simply a manifestation, or an instance of dominating relations that are already in place. Accordingly, subjecting Ms. Carrion to risk, for instance, is derivatively wrong, rather than distinctively wrong because of the relationship she stands in with her risk-imposers is *itself* dominating in nature.

By contrast, if the latter picture is correct, then domination emerges from, or is an upshot of, the imposition of risk itself or because of Ms. Carrion’s (heightened) vulnerability to domination by her employers given that they have uncontrolled or arbitrary capacity to impose risks on her as they please. Accordingly, the wrongness of imposing risks on Ms. Carrion does not derive from the fact that her employers already dominate her, but rather, the wrongness is constitutive of Ms. Carrion being subjected to a form of dominating risk imposition.

In response, we would like to highlight that while our Domination Account explicitly motivates this latter picture, it also allows for the possibility that sometimes, imposition of risk can be distinctively, rather than derivatively wrong even in cases where risk is imposed as part of a pre-existing relation of domination. To explicate this in some detail, we start by distinguishing between two aspects of domination: one, that domination comes in degrees (Pettit 2016: 581) and, two, that domination can be domain-specific (Schmidt 2018).

Regarding the degree aspect, the strength of someone’s dominating power can vary with how much control dominators have over you, the range and extent of one’s option freedoms that are subject to their power to interfere, as well as how effectively or wilfully they will exercise their power. Accordingly, one can be dominated to more or less of a degree, and more or less intensely. Regarding the domain aspect, whilst dominators may enjoy dominating power over one’s option in one (or more) areas or domains of one’s life, they may not enjoy any dominating power over others.

These two aspects allow us to see how risk-bearers like Ms. Carrion can be distinctively wronged *viz-a-viz.* being subjected to dominating risk impositions by her employers with whom she already stands in a relation of domination. First, imposing risks on her can be dominating in and of itself, and thus distinctively wrong, if and when it contributes to *further* intensifying or strengthening the pre-existing relation of domination between the two, and consequently, affecting the degree to which risk-bearers are already dominated. Considered this way, we can even say that part of the explanation of *why* they are in a relationship of domination is *because* the risk-imposers enjoy uncontrolled power over what risks they can expose the risk-bearers to, amongst other things.

Second, imposing risks can be dominating in and of itself if it goes some way towards creating or establishing domination in a distinct domain or sphere of the employee’s life at their workplace wherein her employers previously did not enjoy dominating power. For instance, her employer may already exercise certain degree of control over how many hours she gets paid, whilst extending the scope of this control over a different domain of her workplace life, such as whether her health is protected from risks while she is at her workplace. Accordingly, subjecting individuals to dominating risk impositions can itself serve as a new source of domination, increases the extent to which she is already dominated, and thus constitutes a distinct wrong.

### The Problem of Over-Inclusiveness

According to a second possible objection, our account is over-inclusive. Take an ordinary instance of playing frisbee in the park. Simply by virtue of exercising one’s capacity to play frisbee at one’s own discretion and without the consent of those who are put at the slight risk of getting hurt, you might be said to have wronged them by virtue of subjecting them to a dominating risk imposition. Similarly, consider the ordinary practice of driving. If we all drive cars and, in doing so, subject each other to the risk of driving into each other, then such risk imposition, too, is dominating in nature insofar as our respective exposure to risks is to some extent dependent on each other’s wills (Schmidt 2018). Taken to its limits, our account would have the following unacceptable implication; everyday risk impositions qualify as instances of dominating risk impositions and thus fall under the same category of wrongful risk impositions as *CORONA* and *CARS*.

The potential upshot, then, is that our account problematically broadens the scope of what we intuitively consider wrongful cases of risk imposition. One way of mitigating this concern is to argue that whilst everyday risk imposition may involve some element of domination or dominating-like effects, this gives us a *pro tanto* reason against such risk impositions which is often outweighed by other moral considerations (e.g., the social or individual benefits of permitting driving or playing frisbee in the park) (cf. Hansson 2003). By contrast, we might think that in cases like *CORONA* and *CARS*, concerns regarding domination are not outweighed by other moral considerations, such as any benefits involved for the risk-bearers.

For instance, the fact that Ms. Carrion might benefit from her exposure to risk (say, she will continue to receive payment for her work) or that Mr. O’Polka might eventually reap benefits from the risky testing of self-driving cars in his neighbourhood does not seem to undermine or outweigh their concerns against being non-consensually and arbitrarily made subject to the risks by the dominating risk-imposers. Accordingly, what separates cases like frisbee-playing and driving from cases like *CORONA* and *CARS* on the Domination Account is that the former are only *pro tanto* wrongful, whilst the latter may be all-things-considered wrongful with respect to domination as a wrong-making feature of the risk imposition.

### The Problem of Under-Inclusiveness

Another potential objection is that our account is under-inclusive. That is, it fails to explain a number of intuitively problematic risk-imposition cases involving children or non-human beings. For instance, consider a person who smokes cigarettes in a small apartment shared with their children, thereby exposing them to risk of getting lung cancer. Or consider a real-life example. In 2018, the Chinese researcher He Jiankui claimed to have gene-edited two healthy embryos, resulting in the birth of two baby girls, Lulu and Nana. The genes of these baby girls had been edited to make them resistant to HIV. This might sound like a great thing. But several commentators found this deeply immoral. Julian Savulescu, for example, of the Oxford Uehiro Centre for Practical Ethics – usually associated with arguments in favor of human enhancement – published a press release calling the experiment “monstrous”. Why monstrous? Because:

[g]ene editing is experimental and. .. associated with off-target mutations, capable of causing genetic problems early and later in life, including the development of cancer.. . If the science progressed in the future and off target mutations reduced to acceptable and accurately measurable levels, it might be reasonable to consider first-in-human trials [.. .] in one category of embryos: those with otherwise lethal catastrophic genetic mutations who are certain to die. Gene editing for this group might be *life-saving*; for these current babies, it is only *life-risking*. These healthy babies are used as genetic guinea pigs. This is genetic Russian Roulette^26^

This is a passionate moral objection to the behavior of a scientist putting two small baby girls at risk. But is it a case where the moral concern is best – or most naturally – interpreted as being a moral objection to interfering with the republican freedom of the two babies? Similarly, consider a case involving non-human animals, say, playing Russian Roulette with someone’s pet dog whilst he’s asleep. This too is a case where imposing risk on another – in this case, a pet dog – is morally problematic. But it is not obvious that a domination-oriented analysis applies in such cases^27^.

On Pettit’s account of domination, our moral duties to babies and non-human animals are not most obviously duties to avoid interacting with them in ways that allow them to enjoy independent freedom as non-domination. As he himself explicitly notes, his account of non-domination is an account of how to interact with fellow adults (Pettit 2014: 217). Presumably, this does not mean that Pettit thinks that it is not possible to act immorally towards children or non-human animals. Rather, it means that when some way of interacting with small children is immoral, it is immoral for some other reason than that it fails to give the small children freedom as non-domination (cf. Nyholm 2014).

Some may read this as a charge of under-inclusiveness against our account. In response, we can say two things. Let us first suppose that we would want to analyze all cases of morally problematic risk imposition in the same way, by isolating some general set of features they all have in common. What this means in relation to the picture we have been painting would be something like this. It is not the case that what is morally problematic with imposing risks on others is at bottom, and most importantly, that it always interferes with republican freedom of those others.

Rather, there is some even more general principle or consideration that explains what all cases of morally problematic risk impositions have in common. And that more general moral principle implies that in a sub-set of cases of morally problematic risk imposition, interference with others’ republican freedom is a lower-level moral problem with these instances of morally problematic risk impositions. In other cases, the more general moral principle might imply that some other lower-level moral problem is more closely related to imposing risks on others. In other words, the idea would be that some general moral principle – e.g. a principle against disregarding others’ morally serious interests – explains why it is sometimes the case that it is wrong to impose risks on others because it makes them unfree, but sometimes wrong for other reasons.

Second, we could instead say that in some cases, what is problematic about imposing risks on others is that it interferes with others’ freedom, whereas in other cases, what is morally problematic about imposing risks on others might be that it amounts to, say, a failure to comply with a duty of beneficence or care. In yet other cases, there might be some other thing that is what is morally problematic about imposing the risks in question. Not all cases of risk imposition, according to this pluralistic perspective, should be morally analysed in the same way. This would still be compatible with its being the case that in a significant and large subset of cases of risk impositions, one of the most important moral complaints about these risk impositions is that they are dominating in the sense we have described above.

## Conclusions

In this article, we have proposed a domination-oriented account of what makes certain forms of risk imposition morally problematic by focusing on real-life cases. On this account, imposition of risk is dominating, or has domination-like effects, when and because risk-imposers’ imposing risks on risk-bearers is constitutive of the former exercising their arbitrary power over the range of safe options that the latter are in a position to choose from. To this end, risk-bearers suffer a distinctive form of injustice that existing accounts in the literature fail to fully acknowledge.

We leave a number of related issues unattended to. These include, but are not limited to, clarifying the several distinct ways or mechanisms by which imposition of risk can be dominating or have domination-like effects, identifying other forms of moral and epistemic harms and injustices associated with instances of dominating risk impositions, and discussing concrete proposals for interventions to prevent and remedy such instances. Discussion of these theoretically and practically relevant issues is beyond the scope of this paper. Our hope is that our discussion might stimulate further debate on the topic.

## Footnotes

1 Adapted from (Romero 2018).

2 Adapted from (Eligon and Bowles 2020).

3 As of February 2021, New York’s attorney general Letitia James filed a lawsuit against Amazon over their failure to provide safe health working conditions to their employees amidst Covid-19 pandemic.

4 This question can be distinguished from a more general one, namely, what makes imposition of risk *as such* wrong – over and beyond wrongs that are contingently or incidentally associated with specific features of the case.

5 We say more about domination in Sect. 3.

6 Note that we remain neutral regarding which conception or interpretation of probability is correct.

7 Accounts that we set aside due to space constraints include rights-based approaches (cf. McCarthy 1997), Risk-as-Harm views (Finkelstein 2003, Placani 2016, Oberdiek 2012, 2019), Murphy and Gardoni’s (2007) Capability View, Parr and Slavny’s (2019) Buck Passing View, and Wolff and de-Shalit’s (2003) Disadvantage View, to name a few.

8 In the next section we say more about how our description of this relational wrong differs from these existing accounts.

9 Discussion of these cases is also found in Wolff 2003, Placani 2016, Parr and Slavny 2019, Finkelstein 2003, Lazar 2019, James 2016, Cripps 2013, Kumar 2019 and 2015, Burri 2020.

10 The Interest Account is discussed by other theorists in different guises. For instance, Stephen Perry (2013) draws a distinction between first order interest in avoiding the risked harm and second-order interest in avoiding the risk itself. In contrast, Seth Lazar 2019 focusses on how imposing risks sets back one’s interest in being respected and being secure.

11 For discussions of how other Scanlonian contractualists handle cases of risk in general, cf. Fried (2012), Kumar (2015), Lenman (2008), Frick (2015).

12 For criticism of Oberdiek’s account, cf. Parr and Slavny 2019, Ferretti 2016, Maheshwari 2021.

13 Compossible options are ones that can be conjunctively exercised c.f Carter 2003.

14 Other liberal theorists argue that being free is not only a matter of actual non-interference with one’s options, but also matter of a person’s abilities or capabilities too. c.f. Sen 1999, Kramer 2003, Schmidt 2016.

15 On a relational conception of domination, what counts as domination does not depend on what an agent actually does to others, but rather, it depends on what they could do in virtue of the relation between them (Cf. Katz 2017).

16 Due to space constraints, we leave it open whether, and to what extent appealing to an account of domination other than Pettit’s (e.g., Rawlsian, purely structural, purely outcome-focused, and so on) entail a different (or similar) understanding of the relational wrong involved in imposing risks. Thanks to an anonymous reviewer for highlighting Rawls’s account to us.

17 Moreover, they may also lack control over what could happen to us *once* they are exposed to risk. That is, it is often not in their control – or at least full control that the risked harm does not materialize for them.

18 Cf. https://www.theatlantic.com/technology/archive/2018/03/can-you-sue-a-robocar/556007/.

19 We discuss this point further in Sect. 4.1.

20 We would like to thank an anonymous reviewer for pressing us to clarify this point.

21 Here we follow Frank Lovett’s framework of domination (Lovett 2010).

22 For a general discussion on mutual domination or domination between equals, see Schmidt 2018.

23 For a discussion of compensation and liability in cases involving reciprocal and non-reciprocal risk imposition, cf. Keating 1997 and Fletcher 1972. We thank an anonymous reviewer for these suggestions.

24 By contrast, Oberdiek’s (2012, 2017) account restricted the scope of power exercised in imposing risk to be constitutive of one’s (illegitimate) authority.

25 We would like to thank an anonymous reviewer for pressing us to emphasis this point.

26 http://blog.practicalethics.ox.ac.uk/2018/11/press-statement-monstrous-gene-editing-experiment/ (accessed on August 15, 2021).

27 For relevant discussion, see Paez 2021.
